# Fourth-generation light sources

**DOI:** 10.1107/S2052252523003585

**Published:** 2023-04-27

**Authors:** Henry N. Chapman

**Affiliations:** a Center for Free-Electron Laser Science CFEL, Deutsche Elektronen-Synchrotron DESY, Notkestr. 85, 22607 Hamburg, Germany; b The Hamburg Centre for Ultrafast Imaging, Universität Hamburg, Luruper Chaussee 149, 22761 Hamburg, Germany; c Department of Physics, Universität Hamburg, Luruper Chaussee 149, 22761 Hamburg, Germany

**Keywords:** fourth-generation light sources, X-ray free-electron lasers, synchrotron radiation facilities, multibend achromats, Editorial

## Abstract

New fourth-generation synchrotron radiation facilities bring large gains in X-ray source brightness, but also challenges in making full use of their potentials. Some of these challenges have been faced at X-ray free-electron laser facilities.

On the northern summer solstice of 2016, Max IV opened in Lund, Sweden, the first of a new generation of synchrotron radiation facilities based on the multibend achromat (MBA). This new innovation provides a way to control the trajectories of giga-electron-volt electrons to micrometre precision. The X-ray beam generated from such an improved ‘fourth generation’ storage ring, measured at the experiment 50 m away, is largely spatially coherent because the angular width of the source, as seen from this distance, is exceedingly small – the same reason why starlight is spatially coherent. Basically, the difference between the distances of paths that rays take to reach the observer from one side of the source or the other is less than a wavelength, such that an optical instrument (including diffraction from the eponymous Young’s double slit or its generalization to a macromolecular crystal) does not yield a result any different to that obtained with a single point emitter. Such can easily be the case when the Young’s ‘slits’ or scatterers are placed very close together. Indeed, the coherence of beams from a laboratory source was adequate for von Laue, Freidrich and Knipping to observe the first diffraction from zinc sulfide and for Rosalind Franklin to create Photograph 51 of the B-form of DNA. (Periodic structures allowed many coherence ‘patches’ to contribute to produce a measurable signal.) The new MBA sources fulfil this condition for scatterers placed at any spacing within the entire microradian extent of the beam (thereby dropping the need for periodicity in the sample). In some new source designs and for photon energies softer than about 10 keV, the entire beam is coherent – referred to as diffraction limited. This implies that the source itself is exceedingly bright. And it is brightness (or brilliance as some call it) that truly defines these sources and provides opportunities in all fields of research that synchrotrons cater to today.

The brightness of a source describes how much light it emits per second and unit area into each solid angle and over a particular bandwidth. Given the above definition of spatial coherence, brightness is seen to be proportional to the number of photons per second passing through a ‘coherence volume’ of phase space (of position and momentum). One can strip away unwanted coherence modes by limiting the solid angle and area of the source that contributes to an experiment, such as by collimating the beam as Friedrich and Knipping first did, but it is a fundamental theorem of optics that one can never increase the number of rays per phase space volume. There are many ways to make things worse, but the source brightness dictates the upper limit on how quickly one can undertake a diffraction experiment, measure a spectrum, collect an image or tomogram, or expose a wafer. Brightness thus forms the basis of the comparison of sources, and the user meeting of every synchrotron radiation facility features one or more graphs of this quantity to boast the preeminence of their source or to justify the need for the next upgrade. What is astounding is the plot of the progression of the average brightness of sources since the discovery of X-rays by Röntgen in 1895. Not much happened until 1947 when synchrotron radiation was observed at the General Electric Research Laboratory in Schenectady, New York (Robinson, 2001 ▸). Since the late 1970s there has been a steady exponential growth that has brought 11 orders of magnitude increase in that time. This represents a doubling every 1.4 years, which can be compared with Moore’s law of a doubling of the number of transistors on a chip about every 2 years. Sirius, at the Brazilian Synchrotron Light Laboratory, is currently under commissioning and the ESRF in France has completed its upgrade to its Extremely Brilliant Source (EBS), giving a 30 times increase in brightness (Raimondi *et al.*, 2023 ▸). Other facilities such as the Advanced Photon Source and the Advanced Light Source in the USA, Spring8 in Japan, Diamond Light Source in the UK, and PETRA in Germany, are soon to follow, with even larger gains in brightness.

As in the fields of microlithography (driving Moore’s law) and high-power optical lasers, the exponential growth of the performance of accelerator-based X-ray sources has relied on considerable effort and ingenuity to fuel progress punctuated by breakthroughs such as the MBA. Another key invention, the undulator, combined with optimized storage ring designs in the ‘third-generation’ synchrotron facilities that have served us for the last 30 years. The periodic magnetic structure delivers constructive interference of emission as the electron traverses each period [scaled from centimetres down to ångstroms by virtue of a double whammy of special relativity (Hwu & Margaritondo, 2021 ▸)]. X-ray free-electron lasers built upon this remarkable device by using enough periods that the radiation field influences the trajectories of the electrons (travelling at near light speed) and imprints itself onto their distribution, so that they all radiate in phase. This sets up a positive feedback that amplifies the FEL pulse and gains us yet another huge jump in peak brightness (and coherence) – both due to the increased number of photons, but also now because the X-rays are generated in femtosecond-duration pulses. Once also labelled as fourth-generation sources, current XFELs are more of a bifurcation that coexists with storage rings to expand X-ray science.

What new capabilities will fourth-generation synchrotron radiation sources bring? There is a clear benefit for imaging and tomography, where the speed at which an image is acquired is directly proportional to brightness. Thus, larger volumes (brains, computer chips) can be imaged in reasonable times, and if the sample can handle it, movies of processes in batteries, materials formation and processing, or energy harvesting can be made. Faster detectors and high-resolution X-ray optics are needed. The full coherence of the beam brings to fruition an idea first expressed by David Sayre, of utilizing the ability of crystallography to phase the wavefield scattering from a sample in order to synthesize an image of any object placed in the beam (Sayre, 1980 ▸). The method of ptychography, first developed in the electron microscope, provides an optimal way to recover the wavefield (Pfeiffer, 2018 ▸). These ideas could be driven all the way to atomic resolution – again, if the sample can handle it, or if information can be combined from many objects. The imaging of biological structures such as cells and macromolecules remains limited by radiation damage, and higher brightness just achieves the inevitable degradation sooner, for the same recorded diffraction information. Nevertheless, highly automated sample delivery methods enable rapid surveys of sensitive structures to be conducted over large parameter spaces, including time-resolved measurements, or to capture rare events, as needed to investigate relationships between structure and function.

Unlike previous transitions, many of the challenges faced by fourth-generation synchrotron facilities have solutions developed at the XFELs. Thirty years ago, the performance of beamlines was often limited by the speed of detectors. XFELs were such a startling jump of a billion times in peak brightness that large investments were necessary in high-speed integrating detectors. These are now a great match for the upgraded synchrotrons (Chapman, 2018 ▸) and can consume photons faster than a storage-ring-based source can provide them (Allahgholi *et al.*, 2015 ▸). These detectors produce vast amounts of data, which is recognized as too large to fully archive and in need of new expertise to turn it into knowledge. Techniques such as serial crystallography, born out of the necessity to work with destructive X-ray pulses, are already being adopted at synchrotron facilities to make full use of the source output and provide a paradigm for high-throughput automated experiments (Standfuss & Spence, 2017 ▸). A strong connection between these different ‘fourth generation’ facilities is needed to tackle what are becoming joint challenges. Looking even further ahead, a higher diversity of sources including energy-recovery linacs (Shen, 2001 ▸), and compact accelerators utilizing high-power laser technologies (Graves *et al.*, 2017 ▸), may benefit from such synergies and serve the needs of structural science with both specialized instrumentation for particular types of measurements or test beds for further dev­elopment. **IUCrJ** will continue to chart the exciting outcomes emerging from this and future generations of sources.
